# Heterozygous Loss of KRIT1 in Mice Affects Metabolic Functions of the Liver, Promoting Hepatic Oxidative and Glycative Stress

**DOI:** 10.3390/ijms231911151

**Published:** 2022-09-22

**Authors:** Raffaella Mastrocola, Eleonora Aimaretti, Gustavo Ferreira Alves, Alessia Sofia Cento, Claudia Fornelli, Federica Dal Bello, Chiara Ferraris, Luca Goitre, Andrea Perrelli, Saverio Francesco Retta

**Affiliations:** 1 Department of Clinical and Biological Sciences, University of Torino, 10043 Orbassano (TO), Italy; 2 CCM Italia Research Network, National Coordination Center at the Department of Clinical and Biological Sciences, University of Torino, 10043 Orbassano (TO), Italy; 3 Department of Neurosciences “Rita Levi Montalcini”, University of Torino, 10126 Torino (TO), Italy; 4 Department of Pharmacology, Federal University of Santa Catarina, Florianópolis 88040-900, Brazil; 5 Department of Molecular Biotechnology and Health Sciences, University of Torino, 10126 Torino (TO), Italy; 6 Department of Pharmacology and Physiology, University of Rochester Medical Center, Rochester, NY 14642, USA

**Keywords:** KRIT1/CCM1, FoxO1, hepatic insulin signaling, hepatic glucose metabolism, redox-metabolic interplay, Nrf2, advanced glycation end-products (AGEs), hepatic antioxidant and antiglycative defenses, adaptive redox and metabolic homeostasis

## Abstract

KRIT1 loss-of-function mutations underlie the pathogenesis of Cerebral Cavernous Malformation (CCM), a major vascular disease affecting the central nervous system (CNS). However, KRIT1 is also expressed outside the CNS and modulates key regulators of metabolic and oxy-inflammatory pathways, including the master transcription factor FoxO1, suggesting a widespread functional significance. Herein, we show that the KRIT1/FoxO1 axis is implicated in liver metabolic functions and antioxidative/antiglycative defenses. Indeed, by performing comparative studies in KRIT1 heterozygous (KRIT1^+/−^) and wild-type mice, we found that KRIT1 haploinsufficiency resulted in FoxO1 expression/activity downregulation in the liver, and affected hepatic FoxO1-dependent signaling pathways, which are markers of major metabolic processes, including gluconeogenesis, glycolysis, mitochondrial respiration, and glycogen synthesis. Moreover, it caused sustained activation of the master antioxidant transcription factor Nrf2, hepatic accumulation of advanced glycation end-products (AGEs), and abnormal expression/activity of AGE receptors and detoxifying systems. Furthermore, it was associated with an impairment of food intake, systemic glucose disposal, and plasma levels of insulin. Specific molecular alterations detected in the liver of KRIT1^+/−^ mice were also confirmed in KRIT1 knockout cells. Overall, our findings demonstrated, for the first time, that KRIT1 haploinsufficiency affects glucose homeostasis and liver metabolic and antioxidative/antiglycative functions, thus inspiring future basic and translational studies.

## 1. Introduction

The KRIT1 (Krev interaction trapped 1) gene has been clearly associated with the pathogenesis of Cerebral Cavernous Malformation (CCM), a cerebrovascular disease affecting approximatively 0.5% of the population worldwide and characterized by the formation of abnormally enlarged and leaky capillary channels (caverns), which are referred to as CCM lesions. These can be detected by conventional magnetic resonance imaging (MRI) techniques and occur primarily in the central nervous system (CNS), where they can remain asymptomatic throughout the entire lifetime or unpredictably give rise to a wide spectrum of clinical symptoms, ranging from recurrent headaches to seizures, focal neurological deficits, and fatal intracerebral hemorrhage [1,2]. Besides the CNS, CCM lesions have also been found in other parts of the body, including bone, skin, eyes, and visceral organs, such as the liver, raising the possibility of both CNS and systemic clinical manifestations [3,4,5,6]. CCM disease is of proven genetic origin and may occur in both sporadic (sCCM) and familial (fCCM) forms. The latter is inherited as an autosomal dominant condition with incomplete penetrance and highly variable expressivity, suggesting the involvement of both primary and secondary determinants of pathogenesis [7,8]. Besides KRIT1 (also known as CCM1), whose mutations account for more than 50% of fCCM cases, CCM disease has been associated with mutations in two other genes, CCM2 and PDCD10 (also known as CCM3), which account for about 20% and 10% of the cases, respectively [9]. However, accumulated evidence in endothelial-specific conditional knockout (cKO) mouse models has clearly demonstrated that even the homozygous loss of any of the three known CCM genes is not sufficient to cause the formation of CCM lesions, thus confirming the necessary contribution of additional determinants, including microenvironmental risk factors and genetic modifiers of tissue sensitivity to stressful conditions [8,10,11]. Accordingly, over the last decade, it has clearly emerged that KRIT1 loss-of-function predisposes the development of CCM lesions by exerting pleiotropic effects on key redox-sensitive mechanisms involved in cellular homeostasis and defenses against oxidative stress and inflammation, leading to enhanced endothelial cell susceptibility to oxy-inflammatory insults [8,10,12,13,14]. Specifically, in 2010, we reported, for the first time, that KRIT1 loss-of-function causes a significant increase in intracellular levels of reactive oxygen species (ROS) as well as a downregulation of the forkhead box O1 (FoxO1) transcription factor, a master regulator of superoxide dismutase 2 (SOD2) and antioxidant responses [15]. Subsequently, we showed that loss of KRIT1 function results in distinct pro-oxidant and pro-inflammatory effects, including induction of the redox-sensitive JNK/c-Jun/COX-2 axis [16], upregulation of NADPH oxidase and NF-κB signaling [17,18], and accumulation of ROS-generating dysfunctional mitochondria, due to defective autophagy [19,20,21]. In turn, these effects lead to abnormal adaptive redox responses, including altered expression and activity of transcription factors and enzymes involved in cellular protection against oxidative and glycative stress, which eventually sensitize cells to microenvironmental stress conditions [12]. In particular, we found that KRIT1 deficiency in cellular models causes a chronic upregulation of the transcription factor nuclear factor erythroid 2-related factor 2 (Nrf2), a master regulator of cell antioxidant defenses, and its downstream target glyoxalase-1 (Glo-1), the main detoxifying enzyme for advanced glycation end-products (AGEs) [22,23], as well as an increased S-glutathionylation of redox-sensitive proteins [24], suggesting potential physio-pathological implications.

Overall, besides adding significant pieces to the molecular puzzle underlying CCM disease pathogenesis, these findings also raised the possibility that the effects of KRIT1 loss-of-function have more systemic implications than previously suspected. Accordingly, our previous study demonstrated that KRIT1 heterozygous mice are susceptible to the development of metabolic-related comorbidities associated with chronic oxy-inflammatory conditions, such as atherosclerosis [25]. Among other possibilities, these considerations prompted us to hypothesize that KRIT1 may play a role in metabolic pathways directly related to redox homeostasis and signaling, including those involved in hepatic glucose metabolism and systemic glucose homeostasis. Accordingly, whereas KRIT1 is almost ubiquitously expressed in human organs and tissues, including brain, heart, liver, intestine, skeletal muscle and bone [26,27,28], the maintenance of glucose metabolism and homeostasis involves regulatory proteins linked to KRIT1 functions, such as FoxO1 [15,29]. Indeed, besides playing a critical role in vascular homeostasis and defense against oxidative stress [30,31,32], FoxO1 is also a master regulator of hepatic glucose metabolism and systemic glucose homeostasis through various mechanisms, including modulation of appetite-regulating hypothalamic circuits, intestinal absorption of nutrients, hepatic expression of metabolic enzymes, and synthesis and secretion of insulin by pancreatic β-cells [33,34]. On the other hand, it is noteworthy that high levels of AGEs, such as those occurring in diabetes and defective antiglycative defense conditions, affect the expression and transcriptional activity of FoxO1, contributing to the failure of its protective function and the onset of glucose homeostasis alterations [35,36,37].

In this light, herein we investigated the role of the KRIT1/FoxO1 axis in hepatic glucose metabolism and systemic glucose homeostasis by taking advantage of a KRIT1 heterozygous mouse model, described previously [15,25,38]. Using this animal model, we assessed whether KRIT1 haploinsufficiency affected food intake, systemic glucose disposal, and plasma levels of metabolic hormones, including leptin, insulin, and glucagon. Moreover, we evaluated its impact on FoxO1 expression and phosphorylation levels in the liver, as well as on FoxO1-dependent signaling pathways, such as hepatic insulin signaling, and on key markers of hepatic metabolic processes, including gluconeogenesis, glycolysis, mitochondrial respiration, and glycogen synthesis. Furthermore, we analyzed the basal nuclear levels of Nrf2, which are indicative of its constitutive activation as antioxidant transcription factor by sustained oxidative stress conditions, as well as the hepatic levels of AGEs and AGE receptors, and the specific activity of the AGE detoxifying enzyme Glo-1, which are instrumental for assessing chronic glycative stress conditions. Finally, to assess the presence of a systemic inflammatory status, we assayed plasma levels of major inflammatory cytokines, including interleuchin-1 β (IL-1β), interleuchin-6 (IL-6), interferon-γ (IFN-γ), and tumor necrosis factor-α (TNF-α).

Taken together, the outcomes of these experimental approaches demonstrated that the functional relationship between KRIT1 and FoxO1 has broader physio-pathological implications than previously suspected, including a significant role in metabolic functions and antioxidant/antiglycative defenses of the liver. Interestingly, while providing novel insights into KRIT1 biological functions and consequent implications for new basic research avenues, our findings also raise the possibility that loss of KRIT1 heterozygous function mutations may predispose carriers not only to CCM disease, but also to metabolic comorbidities, thus, opening the door to novel perspectives for future preclinical and clinical studies.

## 2. Results

### 2.1. Heterozygous Loss of KRIT1 in Mice impairs Food Intake, Systemic Glucose Disposal and Plasma Levels of Metabolic Hormones

Heterozygous KRIT1 knockout (KRIT1^+^^/−^) mice and wild-type (WT) littermate controls were fed the same diet and periodically analyzed in parallel up to 6 months (26 weeks) of age, as described in Materials and Methods. At 26 weeks of age, KRIT1^+^^/−^ mice had significantly lower body weight and reduced food intake than corresponding WT littermate controls, as well as significantly lower fasting plasma glucose levels (Table 1). Moreover, the analysis of metabolic hormones showed that fasting plasma levels of leptin and insulin were markedly decreased in KRIT1^+^^/−^ mice as compared to their WT counterparts, while glucagon levels were slightly increased (Table 1).

**Figure 1:**
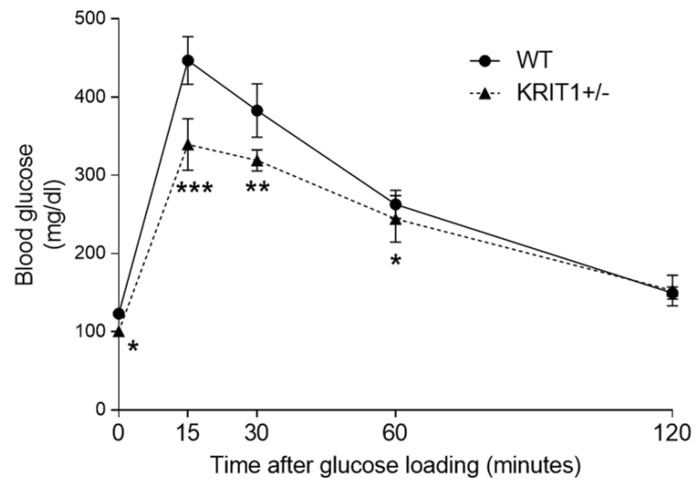
**Glycemia monitoring during oral glucose tolerance test (OGTT).** OGTT was performed in 26-week-old heterozygous KRIT1 knockout (KRIT1^+/−^) mice and wild-type (WT) littermate controls after a fasting period of 18 h by administering a glucose solution (2 g/kg) orally via gavage. Plasma glucose levels were measured with a glucometer before glucose administration (time zero) and 15, 30, 60 and 120 min afterward. Data are means ± SEM. * *p* < 0.05; ** *p* < 0.005; *** *p* < 0.001 vs. WT. Notice that KRIT1^+/−^ mice reached a significantly lower plasma glucose peak level 15 min after glucose loading, compared to the WT littermate controls.

Furthermore, the outcomes of oral glucose tolerance test (OGTT) showed that KRIT1^+/−^ mice reached a markedly lower plasma glucose peak level 15 min after glucose loading, compared to the WT littermate controls, and glucose levels remained significantly lower at all subsequent time points (Figure 1).

### 2.2. Heterozygous Loss of KRIT1 in Mice causes Downregulation of FoxO1 in the Liver

**Figure 2:**
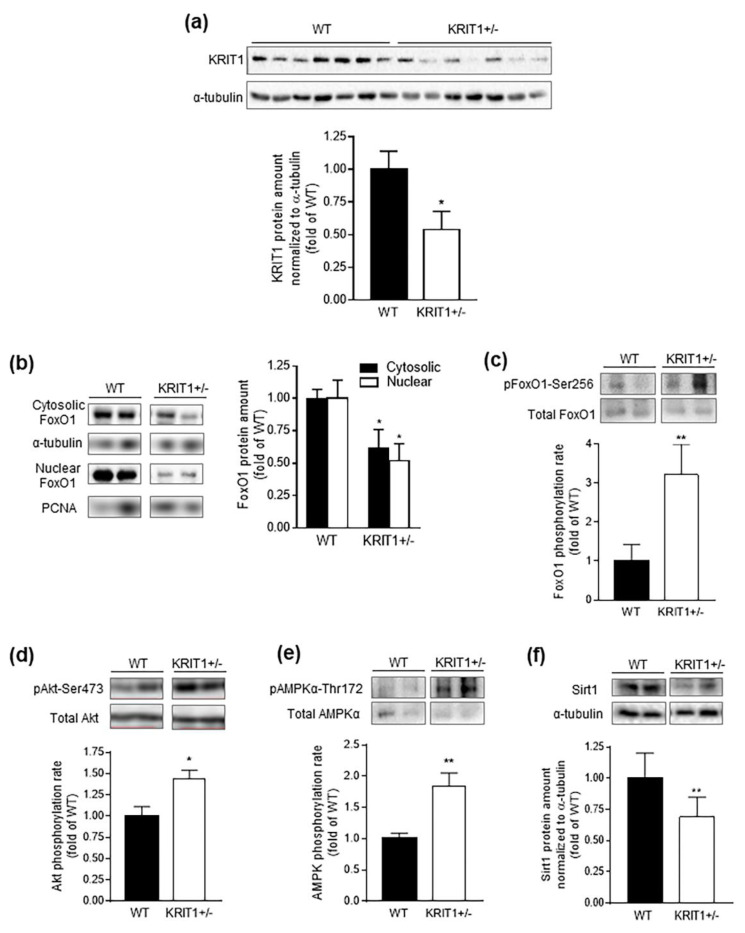
**Heterozygous loss of KRIT1 in mice caused downregulation of FoxO1 in the liver.** (**a**) Representative Western blots showing a downregulation of KRIT1 protein levels in liver homogenates from KRIT1 heterozygous (KRIT1^+^^/−^) mice vs. their wild-type (WT) littermates. (**b**) Representative Western blots showing a significant downregulation of both cytoplasmic and nuclear FoxO1 expression levels in subcellular fractions of liver homogenates from KRIT1^+^^/−^ mice vs. their WT counterparts. Histograms represent the densitometric quantification of FoxO1 bands of 7 mice per group normalized to the corresponding α-tubulin and proliferating cell nuclear antigen (PCNA) loading controls for cytosolic and nuclear fractions, respectively. (**c**) Representative Western blots showing a significant increase in FoxO1 phosphorylation at Ser256 in whole liver homogenates from KRIT1^+^^/−^ vs. WT mice. The histogram represents the densitometric quantification of phosphorylated FoxO1 bands of 7 mice per group normalized to the corresponding total forms. (**c**–**e**) Representative Western blots showing a marked increase in the activating phosphorylation of Akt/protein kinase B (pAkt-Ser473) (**d**) and AMP-activated protein kinase (pAMPK-Thr172) (**e**) serine/threonine kinases, as well as a significant downregulation of the expression level of Sirtuin 1 (Sirt1) deacetylase (**f**) in liver homogenates from KRIT1^+^^/−^ vs. WT mice. Histograms represent the densitometric quantification of pAkt-Ser473, pAMPK-Thr172 and Sirt1 protein bands of 7 mice per group normalized to the corresponding total forms or loading control (α-tubulin), respectively. Data are means ± SEM of 7 mice per group and are presented as fold changes relative to the WT control. * *p* < 0.05; ** *p* < 0.005 vs. WT.

Consistent with distinct reports of a homogeneous expression of KRIT1 in the liver [26,27], including hepatocytes [39], our Western blot analysis detected significant KRIT1 protein levels in liver homogenates from WT control mice. However, these levels were comparatively reduced in KRIT1^+^^/−^ mice (Figure 2a). On the other hand, our previous studies in cellular models demonstrated that KRIT1 influenced, directly and dose-dependently, the expression levels of FoxO1, as well as demonstrating that this relationship was strictly correlated with the maintenance of intracellular ROS homeostasis [15]. Accordingly, FoxO1 is known to be a master transcriptional regulator of cellular responses to ROS and oxidative stress through the induction of genes encoding antioxidant enzymes, including SOD2 and catalase [15,40]. Interestingly, besides its role in antioxidant responses and oxidative stress resistance, it has been established that the FoxO1 transcription factor also plays a key role in insulin signaling and hepatic glucose metabolism [34,41,42], suggesting the possible implication of KRIT1 in critical FoxO1-dependent metabolic pathways in the liver. To address this possibility, we first analyzed FoxO1 expression and phosphorylation levels in the liver of KRIT1^+^^/−^ mice and WT littermate controls (Figure 2b,c). As shown in Figure 2b, Western blot analysis of subcellular fractions of liver homogenates demonstrated a significant downregulation of both cytoplasmic and nuclear FoxO1 protein levels in the liver of KRIT1^+^^/−^ mice as compared to their WT counterparts (Figure 2b). Moreover, Western blot analysis of whole liver homogenates from KRIT1^+^^/−^ mice also showed a significant increase in FoxO1 phosphorylation at Ser256 (Figure 2c), which was indicative of an increase in its multistep negative regulation, including nuclear exclusion and transcriptional activity inhibition [43] and ubiquitination-mediated proteasomal degradation [44]. In addition, to provide further insights into the observed downregulation of FoxO1 in the liver of KRIT1^+^^/−^ mice, we performed Western blot analysis of protein kinase B (PKB)/Akt, AMP-activated protein kinase (AMPK) and Sirtuin 1 (Sirt1), three major regulators of FoxO1 nuclear/cytoplasmic shuttling and transcriptional functions [45,46]. The outcome of these analyses showed a marked increase in the activating phosphorylation of both PKB/Akt (Figure 2d) and AMPK (Figure 2e), two serine/threonine kinases known to modulate FoxO1 transcriptional activity [43,45,47,48]. Specifically, Akt-mediated phosphorylation of FoxO1 induced its nuclear export and retention in the cytoplasm, and consequent transcriptional inactivation, in response to various extracellular stimuli, including insulin and other growth factors, and cytokines [48,49]. FoxO1 phosphorylation by AMPK has, instead, been implicated in adaptive responses to metabolic and oxidative stress conditions, and shown to result in either a short-term compensatory increase or a long-term decrease in FoxO1 transcriptional activity through the induction of its nuclear translocation or proteasomal degradation, respectively [47,48,50,51,52]. Concomitantly, we also observed a significant downregulation of Sirt1 (Figure 2f), a NAD-dependent deacetylase that is known to increase nuclear retention and transcriptional activity of FoxO1 by deacetylating lysine residues in its DNA binding domain [53,54]. Overall, these findings demonstrated that heterozygous loss of KRIT1 in mice affected major regulators of FoxO1 and ultimately led to a significant downregulation of FoxO1 expression and transcriptional activity in the liver.

### 2.3. Heterozygous Loss of KRIT1 in Mice affects Hepatic Insulin Signaling

**Figure 3:**
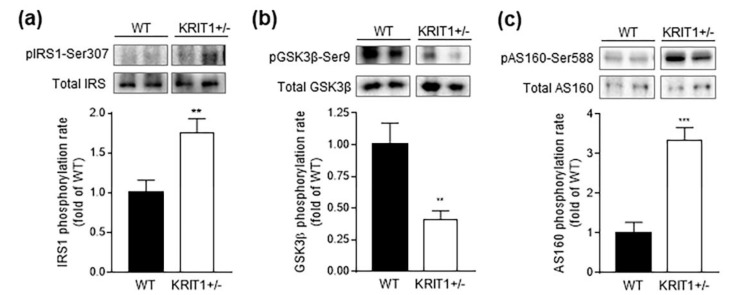
**Heterozygous loss of KRIT1 in mice affected hepatic insulin signaling.** (**a**–**c**) Representative Western blots and related histograms showing significant differences in liver homogenates from KRIT1 heterozygous (KRIT1^+^^/−^) vs. wild-type (WT) mice with respect to phosphorylation level of key regulatory proteins involved in insulin signaling, including a marked increase and decrease in inactivating phosphorylation of insulin receptor substrate 1 (pIRS1-Ser307) (**a**) and glycogen synthase kinase 3β (pGSK3β-Ser9) (**b**), respectively, and an equally marked increase in activating phosphorylation of Akt substrate of 160 kDa (pAS160-Ser588) (**c**). The histograms represent the densitometric quantification of phosphorylated protein bands of 7 mice per group normalized to the corresponding total forms. Data are means ± SEM of 7 mice per group and are presented as fold changes relative to the WT control. ** *p* < 0.005; *** *p* < 0.001 vs. WT.

The observed differences between 26-week-old KRIT1^+^^/−^ mice and their WT counterparts, including the enhanced plasma glucose clearance during OGTT and the marked increase in activating phosphorylation of Akt in the liver, prompted us to test whether the hepatic insulin signaling was affected by KRIT1 haploinsufficiency. To this end, we analyzed the activation status of upstream regulators and downstream targets of Akt involved in insulin signal transduction, such as insulin receptor substrate 1 (IRS1), which links the insulin receptor to PI3K/Akt activation, and glycogen synthase kinase 3β (GSK3β), a substrate of Akt in insulin-stimulated cells [55]. Unexpectedly, we detected an increased inactivation of IRS1 in liver extracts of KRIT1^+^^/−^ mice compared to their WT counterparts, as indicated by the marked increase in IRS1 phosphorylation at Ser307 (Figure 3a), which is known to block IRS1 interaction with the insulin receptor and inhibit insulin stimulation of the PI3K/Akt signaling [56]. Consistent with an impaired insulin signaling, we also detected a marked decrease in GSK3β phosphorylation at Ser9 (Figure 3b), which is known to underlie the inhibition of GSK3β kinase activity by insulin-regulated kinases [57,58]. In addition, we analyzed the phosphorylation status of Akt substrate of 160 kDa (AS160), an established substrate for both Akt and AMPK protein kinases, that is required for insulin-stimulated glucose transporter 4 (GLUT4) translocation to the cell surface, and consequent increase in cellular glucose uptake [59,60]. Specifically, our Western blot analysis of AS160 phosphorylation at Ser588, which is crucial for AS160-mediated GLUT4 translocation, demonstrated a marked increase in phosphorylation levels in the liver of KRIT1^+^^/−^ mice, as compared to WT controls (Figure 3c), suggesting an enhanced hepatic glucose uptake, which was not, however, dependent on insulin signaling. Consistently, there has been evidence for AMPK-dependent GLUT4 upregulation and enhancement of cellular glucose uptake that might occur independently of the insulin signaling under metabolic disorder and oxidative stress conditions [61,62].

### 2.4. KRIT1 Haploinsufficiency affects Major Regulatory Proteins implicated in Hepatic Glucose Metabolism

**Figure 4:**
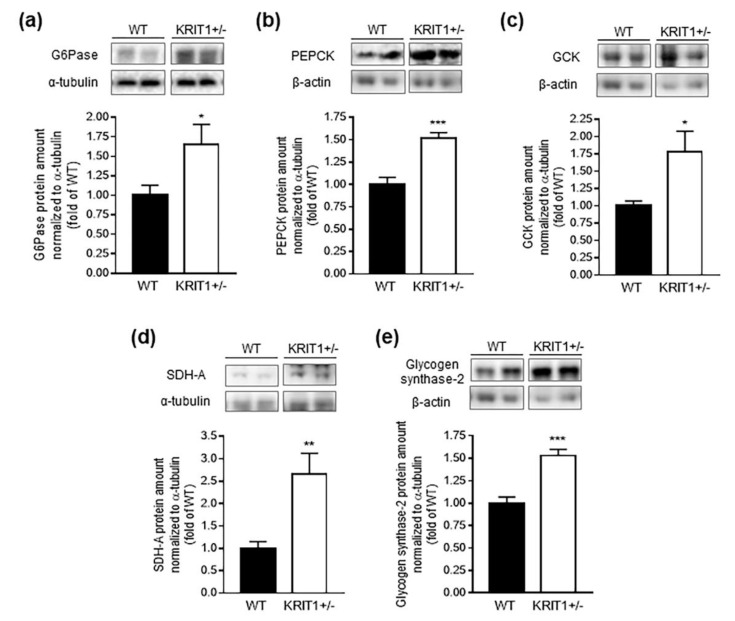
**KRIT1 haploinsufficiency affected major regulatory proteins implicated in hepatic glucose metabolism.** (**a**–**e**) Representative Western blots and related histograms showing the increased expression levels of key enzymes involved in gluconeogenesis [glucose 6-phosphatase (G6Pase) (**a**) and phosphoenolpyruvate carboxykinase (PEPCK) (**b**)], glycolysis [glucokinase (GCK) (**c**)], mitochondrial energy metabolism [succinate dehydrogenase A (SDH-A) (**d**)], and glycogen synthesis [glycogen synthase-2 (GYS2) (**e**)] in liver homogenates from KRIT1^+^^/−^ mice vs. WT littermate controls. The histograms represent the densitometric quantification of protein bands of 7 mice per group normalized to the corresponding loading control (α-tubulin). Data are means ± SEM of 7 mice per group and are presented as fold changes relative to the WT control. * *p* < 0.05; ** *p* < 0.005; *** *p* < 0.001 vs. WT.

Our findings that heterozygous loss of KRIT1 in mice caused both a significant downregulation of FoxO1 and an altered phosphorylation of regulatory proteins associated with insulin signaling and glucose uptake in the liver, including Akt, AMPK, IRS1, GSK3β and AS160, suggested a potential impairment of hepatic glucose metabolism. To address this possibility, we assessed the relative expression level of key enzymes involved in gluconeogenesis, glycolysis, and glycogen synthesis by comparative Western blot analysis of whole liver homogenates from KRIT1^+^^/−^ mice and WT littermate controls (Figure 4). Surprisingly, despite the significant downregulation of FoxO1 and potential increase in glucose disposal observed in the liver of KRIT1^+^^/−^ mice, we found an upregulated expression of the gluconeogenic enzymes glucose 6-phosphatase (G6Pase) (Figure 4a) and phosphoenolpyruvate carboxykinase (PEPCK) (Figure 4b), two established transcriptional targets of FoxO1 in hepatocytes and the rate-limiting enzymes in hepatic gluconeogenesis [63]. Moreover, this apparently paradoxical activation of gluconeogenesis in the liver of KRIT1^+^^/−^ mice was paralleled by an increased expression of glucokinase (GCK) (Figure 4c), the predominant hexokinase isoenzyme in the liver, which catalyzes the phosphorylation of glucose to glucose-6-phosphate, the first and rate-limiting step for both glycolysis and glycogen synthesis [64]. Consistent with an increased glycolytic flux, we found a correlation with enhanced expression levels of succinate dehydrogenase A (SDH-A) (Figure 4d), a catalytic subunit of the mitochondrial respiratory complex II involved in both the Krebs cycle and the electron transport chain [65], suggesting a potential concomitant stimulation of hepatic mitochondrial energy metabolism. Furthermore, consistent with a potential increase in both glucose uptake and gluconeogenesis, as well as with the dominant control on glycogen synthesis and glycolysis exerted by GCK [64], a markedly increased expression of glycogen synthase-2 (GYS2) was also detected (Figure 4e). While these results were mainly based on analysis of protein expression levels, which might not always reflect protein activity, there was clear evidence that expression and activity of enzymes involved in major metabolic pathways, such as glycolysis and mitochondrial respiration, were fully correlated [66,67]. Taken together, these findings suggested that KRIT1 haploinsufficiency affected major regulatory proteins implicated in various pathways of hepatic glucose metabolism, including glucose uptake, gluconeogenesis, glycolysis, and glycogenesis, leading to abnormal adaptive changes in glucose homeostasis that might predispose the development of chronic metabolic hepatic disorders and pro-inflammatory states.

### 2.5. Heterozygous Loss of KRIT1 in Mice alters Hepatic Antioxidant Systems

Alterations in hepatic glucose metabolism and mitochondrial oxidative phosphorylation are tightly interconnected with changes in redox homeostasis and signaling, and may result in decreased hepatic antioxidant defenses and increased oxidative stress [68,69]. Indeed, whereas the rates of aerobic metabolism and ROS production are positively correlated, the intimate liaison between redox and metabolic pathways is highlighted by their sharing of master regulators, including FoxO1 [33,40]. Therefore, we addressed whether the observed alterations in hepatic glucose metabolism caused by heterozygous loss of KRIT1 in mice were associated with changes in major determinants of hepatic antioxidant defenses, including Nrf2, the master regulator of the antioxidant transcriptional response [70], and glutathione (GSH), the predominant antioxidant molecule and main regulator of cellular redox status in the liver [71].

**Figure 5:**
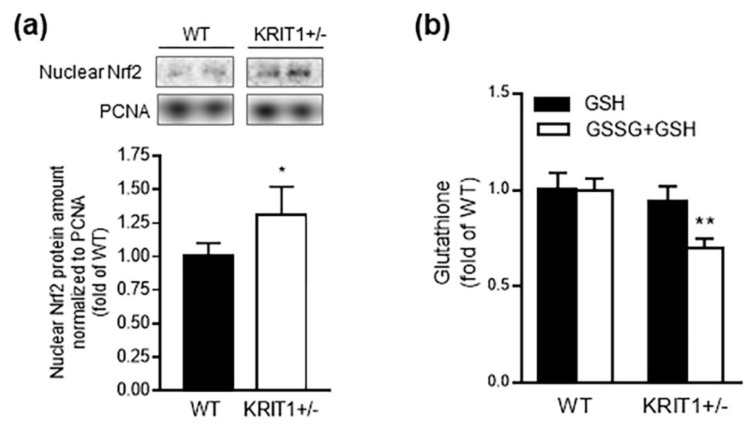
**Heterozygousloss of KRIT1 in mice altered hepatic antioxidant systems.** (**a**) Representative Western blot analysis of liver homogenates showing an increased nuclear accumulation of the antioxidant transcription factor Nrf2 (nuclear factor erythroid 2-related factor 2) in the liver of KRIT1^+^^/−^ vs. WT mice. The histogram represents the densitometric quantification of Nrf2 bands of 7 mice per group normalized to the corresponding loading control [proliferating cell nuclear antigen (PCNA)]. (**b**) Analysis of reduced glutathione (GSH) and total glutathione (GSSG + GSH) levels in liver homogenates from WT and KRIT1^−^^/−^ mice. Data are means ± SEM of 7 mice per group and are presented as fold changes relative to the WT control. * *p* < 0.05; ** *p* < 0.005 vs. WT.

Consistent with our previous findings in KRIT1 knockout cellular models and surgical samples of human CCM lesions [22,23], Western blot analysis of subcellular fractions of liver homogenates demonstrated an increased nuclear accumulation of Nrf2 in the liver of KRIT1^+^^/−^ mice as compared to their WT counterparts (Figure 5a), suggesting the induction of a sustained adaptive antioxidant response. However, this adaptive response was not sufficient to restore a normal redox steady state, as demonstrated by the reduced levels of total GSH detected in the liver of KRIT1^+^^/−^ as compared to WT mice (Figure 5b), which was also consistent with our previous findings in cellular models [12,24], suggesting an impairment in redox homeostasis and antioxidant capacity.

### 2.6. Heterozygous Loss of KRIT1 in Mice alters AGE Accumulation and Expression of AGE Receptors and Detoxifying Systems

**Figure 6:**
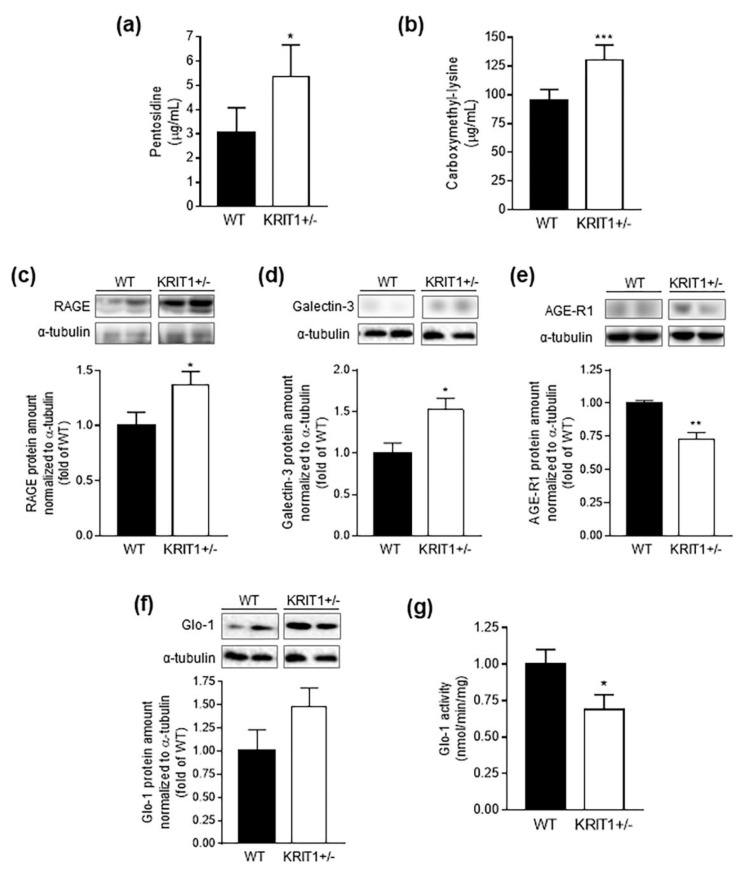
**Heterozygous loss of KRIT1 in mice altered AGE accumulation and expression of AGE receptors and detoxifying systems in the liver.** (**a**,**b**) Concentrations of two major types of AGEs, pentosidine (**a**) and Nε-carboxymethyl-lysine (**b**), quantified by ultraperformance liquid chromatography-tandem mass spectrometry (UPLC-MS) analysis of liver homogenates from WT and KRIT1^+^^/−^ mice. (**c**–**e**) Representative Western blots showing the upregulation of pro-inflammatory AGE receptors, such as the receptor for AGE (RAGE) (**c**) and galectin-3 (Gal-3) (**d**), the downregulation of the anti-inflammatory receptor AGE-receptor-1 (AGE-R1) (**e**), and the upregulation of the AGE detoxifying enzyme glyoxalase-1 (Glo-1) (**f**) in the liver of KRIT1^+^^/−^ vs. WT mice. The histograms represent the densitometric quantification of protein bands of 7 mice per group normalized to the corresponding loading control (α-tubulin). (**g**) Enzymatic activity of Glo-1 in liver homogenates from WT and KRIT1^+^^/−^ mice, determined spectrophotometrically by measuring the increased absorbance at 240 nm due to the catalyzed formation of S-D-lactoylglutathione from methylglyoxal (MG)-GSH hemithioacetal adducts. Data are means ± SEM of 7 mice per group and are presented as the fold change in Glo-1 enzymatic activity relative to the WT control. * *p* < 0.05; ** *p* < 0.005; *** *p* < 0.001 vs. WT.

Among the various Nrf2 target genes implicated in cellular defense against toxic and oxidative insults, there is Glo-1, a major GSH-dependent enzyme involved in the detoxification of AGEs, toxic compounds formed both in vivo and ex vivo during the Maillard reaction between glucose and the amino group of proteins, and associated with an increase in oxidative stress [72,73]. Given our previous findings that a sustained Nrf2-mediated adaptive antioxidant response did not rescue the impairment in redox homeostasis and antioxidant capacity caused by KRIT1 deficiency both in cellular models [22,23,24] and in the liver of KRIT1^+^^/−^ mice (Figure 5a,b), and considering the potential increase in cellular glucose availability and glycolytic flux, we then evaluated AGE levels and AGE receptors and detoxification systems in the liver of KRIT1^+^^/−^ mice (Figure 6). We found that levels of pentosidine (PEN) and Nε-carboxymethyl-lysine (CML), two major classes of AGEs that serve as markers of AGE accumulation in several tissues, were significantly higher in the liver of KRIT1^+^^/−^ as compared to WT mice (Figure 6a,b), an effect likely linked to a related increase in glucose disposal and glycolytic flux. In particular, we detected significant alterations in the expression level of major AGE receptors, including a marked upregulation of the receptor for AGE (RAGE) (Figure 6c) and galectin-3 (Gal-3) (Figure 6d), which are known to generate pro-oxidant and pro-inflammatory effects [74,75,76], and a downregulation of the AGE-degrading receptor AGE-receptor-1 (AGE-R1) (Figure 6e), which is instead endowed with anti-inflammatory and antioxidant activities [77]. In addition, consistent with our previous findings in KRIT1 knockout cellular models [22,23], we found a significant increase in the expression level of the Glo-1 enzyme (Figure 6f), which was likely part of the sustained Nrf2-mediated adaptive antioxidant response. However, specific enzymatic assays showed that the adaptive increased expression of Glo-1 did not correlate with a corresponding increase in its enzymatic activity, as this was markedly depleted in the liver of KRIT1^+^^/−^ when compared with WT mice (Figure 6g), possibly due to a reduced availability of its cofactor GSH, suggesting an ineffective adaptation and a potential contribution to the observed accumulation of AGEs.

Consistent with the potential pro-inflammatory effects, following the observed upregulation of RAGE and Gal-3 and downregulation of AGE-R1, the analysis of plasma cytokine levels revealed a small, but significant, increase in circulating level of major pro-inflammatory markers, such as IL-1β and TNF-α, in KRIT1^+^^/−^ compared to WT mice (Table 2), suggesting the presence of a chronic low-grade systemic inflammatory status. Such chronic condition was further supported by a significant decrease in circulating levels of IL-6 (Table 2), a cytokine known to exert anti-inflammatory activities by controlling the level of pro-inflammatory cytokines [78], and the deficiency of which has been correlated with signs of liver inflammation [79].

### 2.7. Alterations in AGE Receptors and Regulatory Proteins of Glucose Metabolism Detected in the Liver of KRIT1^+/−^ Mice Are recapitulated in KRIT1 Knockout Cellular Models

**Figure 7:**
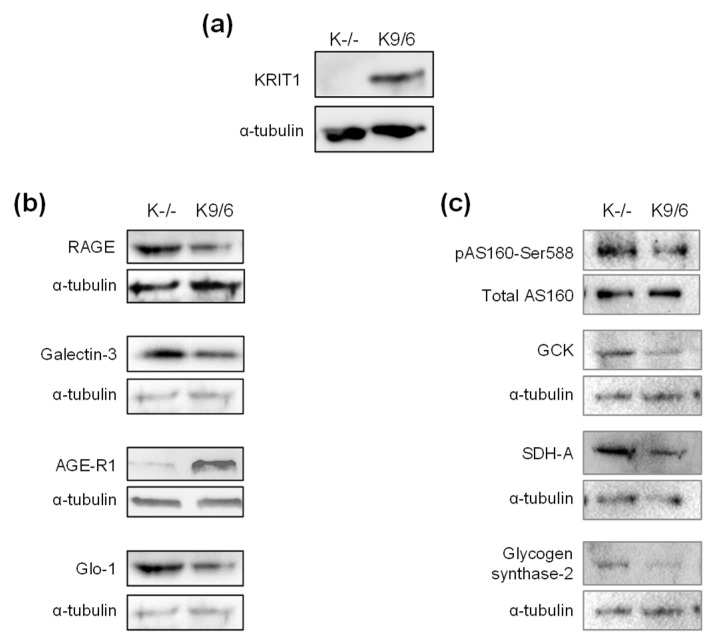
**Alterations in AGE receptors and regulatory proteins of glucose metabolism detected in the liver of KRIT1^+/−^ mice were recapitulated in KRIT1 knockout cellular models.** (**a**) Representative Western blot of KRIT1 protein expression in KRIT1 knockout (K-/-) and KRIT1 re-expressing (K9/6) MEF cells. (**b**) Representative Western blots showing the upregulation of receptor for AGE (RAGE) and galectin-3 (Gal-3), the downregulation of AGE-receptor-1 (AGE-R1), and the upregulation of glyoxalase-1 (Glo-1) expression levels in KRIT1 knockout (K-/-) vs. KRIT1 re-expressing (K9/6) MEF cells. (**c**) Representative Western blots showing the upregulation of markers of glucose uptake [phosphorylated Akt substrate of 160 kDa (pAS160-Ser588)], glycolysis [glucokinase (GCK)], mitochondrial energy metabolism [succinate dehydrogenase A (SDH-A)], and glycogen synthesis (glycogen synthase-2) in KRIT1 knockout (K-/-) vs. KRIT1 re-expressing (K9/6) MEF cells. The level of α-tubulin has been used as loading control. Data are representative of 4 independent experiments.

To confirm the causal relationship between KRIT1 deficiency and the alterations in AGE receptors and regulatory proteins of glucose metabolism observed in the liver of KRIT1^+^^/−^ mice, we extended the investigation to KRIT1 knockout (K-/-) and KRIT1 re-expressing (K9/6) MEF cells (Figure 7a), two established cellular models with an homogeneous genetic background that allow the identification of new molecules and mechanisms involved in KRIT1 physio-pathological functions [38]. As shown in Figure 7, we found that both the upregulation of the pro-inflammatory AGE receptors RAGE and Gal-3, and the downregulation of the detoxifying anti-inflammatory receptor AGE-R1, were recapitulated in K-/- as compared to K9/6 MEF cells, along with the upregulation of Glo-1 (Figure 7b), suggesting that they were indeed KRIT1-dependent effects. Furthermore, whereas the increased phosphorylation of Akt and marked downregulation of FoxO1 in K-/- as compared to K9/6 MEF cells were previously reported [15], we found that these effects were accompanied by the same alterations in the expression level of markers of glucose uptake, glycolysis, mitochondrial respiration and glycogen synthesis detected in the liver of KRIT1^+^^/−^ mice (Figure 3c and Figure 4c–e), including a marked increase both in AS160 phosphorylation and in expression levels of GCK, SDH-A and glycogen synthase-2 enzymes (Figure 7c), suggesting that these effects were also linked to KRIT1 deficiency.

## 3. Discussion

The KRIT1 gene is widely expressed in human and mouse tissues, including in the nervous system, the thymus, the epidermal, digestive, respiratory, uterine and urinary epithelia, and ossification centers, indicating a widespread functional significance, not restricted to the cerebrovascular system [26,27,28]. However, extracerebral phenotypes of KRIT1 loss-of-function mutations have been poorly investigated in in vivo models.

Recently, we reported that heterozygous KRIT1 knockout (KRIT1^+/−^) mice exposed to a high-fructose diet, known to induce systemic oxidative stress and inflammation, had an approximately 2-fold enhanced fat accumulation in the aortic arch and aortic root, as compared to corresponding wild-type (WT) littermates fed the same high-fructose diet, suggesting that KRIT1 may be implicated in intermediary metabolism–redox circuits underlying atherosclerosis development [25].

In the present study, we demonstrate, for the first time, that heterozygous loss of KRIT1 in mice has a major impact on the liver, causing significant alterations in its metabolic and redox functions, promoting oxidative and glycative stress conditions, and affecting systemic glucose homeostasis and inflammatory profile.

Since its original identification in 1999 as the major gene responsible for CCM disease [80,81], KRIT1 functional and dysfunctional effects have been studied mainly in vascular tissues and endothelial cells. Indeed, whereas the constitutive homozygous deletion of KRIT1 in mice was shown to result in embryonic lethality by gestational day E-9.5 due to cardiovascular defects [82], key discoveries in cellular models and endothelial-specific cKO mice have revealed that loss of KRIT1 function in endothelial cells caused a plethora of dysfunctions in various molecular mechanisms involved in vascular homeostasis and disease [13]. Among the pleiotropic effects of loss of KRIT1 function, growing evidence has accumulated over the past decade providing strong support for a major implication of alterations in key redox-dependent mechanisms involved in cellular homeostasis and defenses against oxidative stress and inflammation, including autophagy and signaling pathways mediated by redox-sensitive transcription factors and regulatory enzymes, such as FoxO1, c-Jun, NF-κB, Nrf2, and PKC [15,16,17,19,23,83]. Consistently, distinct pathological phenotypes associated with loss of KRIT1 function in experimental models could be prevented or rescued by either targeted antioxidant enzymes or potential pharmacological compounds endowed with antioxidant, anti-inflammatory and/or pro-autophagic activities, such as Tempol, vitamin D, rapamycin, and avenanthramides; thus, providing further support to a key contribution in oxy-inflammatory mechanisms [10,12,14,17,19,22,84,85,86,87,88].

While highlighting the complex nature of KRIT1 physio-pathological functions in cerebrovascular tissues, investigations in KRIT1 knockout or silenced cellular models and endothelial-specific cKO mice were mainly focused on identifying molecular mechanisms and therapeutic targets for CCM disease, only marginally considering the emerging potential widespread roles of KRIT1. Indeed, inducible KRIT1 knockout mouse models, generated by Cre recombinase technology, have proven useful for understanding mechanisms of CCM lesion genesis and testing therapeutics, as CCM lesions develop reproducibly around postnatal day 6 (P6) upon tamoxifen-mediated induction of KRIT1 deletion at P1 [89,90]. However, CCMs are mainly restricted to the hindbrain and retina, where angiogenesis persists until P10, and tamoxifen treatment after P10 does not result in CCM lesion formation, suggesting that homozygous loss-of-function of KRIT1 only predisposes to CCM pathogenesis, which requires the additive effect of microenvironmental determinants, including local pro-angiogenic conditions, and is influenced by genetic modifiers [7,8,91]. Consistently, studies in the KRIT1^+/−^ mouse model, generated by conventional homologous recombination and mimicking of the heterozygous state of fCCM patients, demonstrated that KRIT1 haploinsufficiency resulted in alterations in redox mechanisms that increase susceptibility to inflammation-induced blood–brain barrier (BBB) leakage and breakdown [17]. Furthermore, this animal model has also allowed the identification of systemic effects caused by loss of heterozygous function mutations in KRIT1, including enhanced susceptibility to endothelial dysfunction and atherosclerosis [25]. These findings prompted us to investigate the possibility that heterozygous loss of KRIT1 function mutations affect organs in which redox mechanisms play a key physio-pathological role, including the liver [92]. Indeed, the highly intertwined relationship between redox state and intermediary metabolism plays a strategic role in the physiopathology of this major metabolic organ [93].

Taking advantage of the KRIT1^+/−^ mouse model [15,25], we found that KRIT1 haploinsufficiency caused a significant downregulation of FoxO1 expression and transcriptional activity in the liver, which was correlated with the modulation of major upstream regulators, such as Akt, AMPK and Sirt1. In turn, these effects were accompanied by alteration of critical FoxO1-dependent metabolic processes in the liver, including insulin signaling and glucose metabolism, as well as by changes in markers of hepatic oxidative and glycative stress. In particular, the significant downregulation of FoxO1 protein levels detected in the liver of KRIT1^+/−^ mice vs. WT littermates was accompanied by a significant increase in FoxO1-Ser256 phosphorylation, which is known to result in the inhibition of its transcriptional activity [43]. Moreover, it was associated with substantial changes in three major regulators of FoxO1 nuclear/cytoplasmic shuttling and transcriptional activity, including enhanced activating phosphorylation of Akt and AMPK serine/threonine kinases, and reduced expression of Sirt1 deacetylase. In turn, these changes are known to have a significant impact on the intricate combination of post-translational modifications (PTMs) underlying the fine-tuned regulation of FoxO1 activity in response to extracellular signals and metabolic and oxidative stress conditions [94]. Interestingly, the signaling pathways involving Akt, AMPK and Sirt1 are highly interconnected and sensitive to the cellular metabolic and redox state, as well as intricately implicated in the regulation of autophagy [95], suggesting potential relationships with the pleiotropic effects of altered redox homeostasis and signaling so far associated with loss of KRIT1 function [8,10,12]. In particular, there is evidence that oxidative inactivation of PTEN, a major negative regulator of PI3K-dependent Akt signaling, causes hyperactivation of Akt and consequent modulation of its downstream targets, including activation of mTOR and inhibition of FoxO1, leading to defective autophagy and enhanced cell sensitivity to oxidative stress [96,97,98]. In this regard, it is noteworthy that both mTOR activation and FoxO1 inhibition were originally linked to loss of KRIT1 function and shown to be associated with increased cellular ROS levels and susceptibility to oxidative stress via downregulation of autophagy and antioxidant defenses [15,19]. On the other hand, both AMPK and Sirt1 are known to play a positive role in the maintenance of cellular homeostasis and antioxidant defenses by opposing the actions of the Akt/mTOR pathway and promoting autophagy [95,99,100]. However, while AMPK and Sirt1 cooperate in orchestrating FoxO1-mediated compensatory responses to transient metabolic and oxidative stress for the maintenance of cell functions, these responses may result in inefficiency in chronic stress conditions. Indeed, there is evidence that sustained activation of AMPK may ultimately lead to FoxO1 proteasomal degradation [47,52], which is also consistent with the reported inactivation of Sirt1 under persistent metabolic and oxidative stress conditions in the liver [101]. Furthermore, sustained activation of AMPK may also result in nuclear accumulation of Nrf2 [102], an adaptive response to altered redox homeostasis that we have previously associated with loss of KRIT1 function in cellular models and surgical samples of CCM disease [22,23], and which we have now found to occur in the liver of KRIT1^+/−^ mice as well. While the precise mechanisms linking KRIT1 haploinsufficiency to the observed changes in Akt and AMPK activating phosphorylation, and Sirt1 expression remain to be fully elucidated. Our findings open a new avenue for future research aimed at addressing the emerging complexity of KRIT1 physio-pathological functions, including its novel implication in intermediary metabolism–redox circuits in the liver.

Remarkably, the downregulation of FoxO1 associated with KRIT1 haploinsufficiency might underlie the reduced food intake and body weight detected in KRIT1^+/−^ mice compared to WT littermate controls fed the same diet, as there is evidence that FoxO1 mediates the action of insulin and leptin in the hypothalamus, thereby controlling mouse feeding behavior [103]. Moreover, the significant downregulation of FoxO1 expression and activity found in the liver of KRIT1^+/−^ mice may account for the observed alterations in hepatic metabolic pathways, including gluconeogenesis, glycolysis, mitochondrial respiration, and glycogen synthesis, as well as for the consequent impact on systemic glucose homeostasis. Such effects are in fact consistent with the key role exerted by FoxO1 in hepatic and systemic glucose metabolism [34,41,42]. In particular, FoxO1 is an established distal effector of insulin signaling through the IRS1/PI3K/Akt pathway, and acts as a negative transcriptional regulator of insulin sensitivity in liver, adipocytes, and pancreatic beta-cells [104,105]. Indeed, either haploinsufficiency or liver-specific inactivation of FoxO1 have been shown to inhibit gluconeogenesis and reduce fasting hyperglycemia in insulin-resistant mice by decreasing hepatic expression of gluconeogenic genes and improving peripheral glucose tolerance [104,105]. Similarly, the liver-specific ablation of FoxO1 was shown to mitigate stress-induced hyperglycemia and hyperinsulinemia, indicating an improvement of systemic insulin sensitivity upon FoxO1 inactivation [106].

Consistent with the established role of FoxO1 in insulin signaling and glucose homeostasis, we found that the downregulation of FoxO1 in the liver of KRIT1^+/−^ mice was associated with enhanced glucose tolerance, as demonstrated by the markedly lower glycemic response curve in the OGTT. Moreover, it also correlated with a significant increase in the activating phosphorylation of AS160, a direct substrate of Akt that is responsible for GLUT4 translocation to the cell surface [59,107], suggesting a stimulation of glucose uptake. Interestingly, these markers of improved glucose uptake seem not to be dependent on activation of the canonical insulin signaling cascade IRS1/Akt/GSK-3β, since we found both lower insulin levels and impaired activation of hepatic IRS1 and GSK-3β proteins in KRIT1^+/−^ mice compared to WT littermate controls. However, consistent with our original discovery in KRIT1 knockout cellular models [15], we found a significant increase in the activating phosphorylation of Akt in the liver of KRIT1^+/−^ mice, suggesting that in this model Akt activation and consequent activating phosphorylation of AS160 may occur independently of insulin stimulation. Besides Akt, AMPK may also contribute to the observed enhanced activating phosphorylation of AS160, which is indeed reported to be a specific target of both Akt and AMPK serine-threonine kinases in the regulation of GLUT4-mediated glucose uptake in the liver [47,108].

In physiological conditions, insulin-induced activation of Akt in response to high glucose levels caused inactivation of FoxO1 and consequent reduced expression of gluconeogenic enzymes, and increased metabolism of glucose via glycolysis and glycogenesis [109]. However, the significant increase in Akt activation and FoxO1 inactivation detected in the liver of KRIT1^+/−^ mice did not correlate with a corresponding increase in insulin levels and canonical signaling, which were even downregulated, suggesting that it occurred as a result of other regulatory mechanisms, including the redox-dependent mechanisms mentioned above. On the other hand, consistent with known effects of FoxO1 downregulation, we found that the liver of KRIT1^+/−^ mice was characterized by significant change in the expression level of major regulatory proteins involved in hepatic glucose metabolism. In particular, we detected an increased expression of glucokinase (GCK), the liver hexokinase enzyme that catalyzes the first step of glycolysis and glycogenesis [64], as well as of succinate dehydrogenase A (SDH-A), a catalytic subunit of the mitochondrial respiratory complex II involved in the Krebs cycle and electron transport chain [65], suggesting an upregulation of hepatic glycolytic flux and mitochondrial respiration. Moreover, we also detected a markedly increased expression of glycogen synthase-2, a key enzyme in glycogenesis, which was likely a compensatory response to an increased glucose uptake leading to glucose storage as glycogen. However, in contrast with known effects of FoxO1 downregulation, we found that glucose 6-phosphatase (G6Pase) and phosphoenolpyruvate carboxykinase (PEPCK), the rate-limiting enzymes of gluconeogenesis and two recognized transcriptional targets of FoxO1 in hepatocytes [63], were upregulated in the liver of KRIT1^+/−^ mice, which was likely to further contribute to the increased intracellular pool of glucose. While further studies are required to address the mechanisms underlying the apparently paradoxical activation of gluconeogenesis in the liver of KRIT1^+/−^ mice, a possible explanation could be related to the observed marked decrease in plasma levels of insulin, which might stimulate the mobilization of the intracellular glucose storage, as previously suggested [110]. Consistent with this possibility, a complementary explanation could arise from our discovery that Sirt1 expression levels were significantly reduced in the liver of KRIT1^+/−^ mice. Indeed, whereas Sirt1 deacetylates FoxO1 to increased its nuclear retention and transcriptional activity [53,111], its reduced expression might contribute not only to the downregulation of FoxO1 detected in the liver of KRIT1^+/−^ mice, but also to the corresponding decrease in insulin plasma levels and increase in markers of enhanced glycolysis and gluconeogenesis. Accordingly, it is known that Sirt1 either suppresses glycolysis by decreasing the expression of GCK or negatively regulates gluconeogenesis by decreasing the expression of G6Pase and PEPCK, depending on the fasting or feeding state, respectively, and enhances insulin secretion from pancreatic beta cells [112].

An increase in intracellular glucose level and flux through glycolysis may result in accumulation of intermediate byproducts, such as diacylglycerol, dihydroxyacetone phosphate and glyceraldehyde phosphate, giving rise to glyoxal and methylglyoxal, the major dicarbonyl precursors of AGEs [113]. Moreover, besides being metabolized through glycolysis and glycogen synthesis, the excess of intracellular glucose is diverted to other metabolic pathways, such as the polyol pathway. This pathway converts glucose to fructose and is typically involved in the production of AGEs, as fructose and its metabolites are more potent nonenzymatic glycation agents than glucose, as well as in the direct induction of oxidative stress through a substantial depletion of NADPH and consequent decrease in the GSH level [114]. In turn, AGEs exert detrimental effects, either directly or via specific AGE receptors [115], and contribute significantly to oxidative stress [114]. Interestingly, although our KRIT1^+/−^ mouse model did not show hyperglycemia, the increased expression of key markers of enhanced glucose uptake and glycolysis/mitochondrial respiration indicated that glucose metabolism was likely to be accelerated and paralleled by perturbed and deregulated gluconeogenesis, thus leading to the same exacerbated metabolism of intracellular glucose observed during diabetes, with consequent accumulation of AGEs.

Consistent with an oxidative stress-like condition resulting from altered glucose metabolism, we found that the accumulation of AGEs in the liver of KRIT1^+/−^ mice was paralleled by changes in major determinants of hepatic antioxidant defenses, including an increased activation of Nrf2, the master transcriptional regulator of cellular antioxidant responses [70]. Notably, while limiting oxidative stress and maintaining an adaptive homeostatic state, such Nrf2-mediated antioxidant responses were ineffective in rescuing basal redox homeostasis in the liver of KRIT1^+/−^ mice, as demonstrated by the presence of reduced levels of glutathione (GSH), the major antioxidant and redox regulator in cells [71], which was consistent with our previous findings in cellular models [22,23]. As a confirmation of the enhanced transcriptional activity of Nrf2 in the liver of KRIT1^+/−^ mice, we found a significant upregulation of the expression level of Glo-1, a Nrf2 transcriptional target implicated in cellular defense against glycative and oxidative stress, acting as the major detoxifying enzyme for AGEs [12,116]. However, the increased expression of Glo-1 was not paralleled by a corresponding increase in its enzymatic activity, which was instead reduced. Such reduction might be explained by a reduced availability of its cofactor GSH, as reported previously [117], suggesting an ineffective adaptive response and a potential contribution to the accumulation of AGEs.

Interestingly, further support to the metabolic and redox alterations and consequent impaired cellular defenses against glycative and oxidative stress detected in the liver of KRIT1^+/−^ mice was provided by the finding that they were accompanied by significant changes in the expression level of major AGE receptors. In particular, we found a marked upregulation of RAGE and Gal-3, two pro-inflammatory AGE receptors, known to induce pro-oxidant and pro-inflammatory effects [77], and a downregulation of the AGE-degrading receptor AGE-R1, which exerts anti-inflammatory and antioxidant activities [77], indicating a pro-inflammatory imbalance. Consistently, the analysis of plasma cytokine levels revealed a small, but significant, increase in circulating levels of major pro-inflammatory markers, such as IL-1β and TNF-α, in KRIT1^+/−^ compared to WT mice, suggesting the presence of a low-grade systemic inflammatory state, which was likely related to the dysregulated hepatic glucose metabolism and inefficient antioxidant and antiglycative defenses. Such chronic low-grade inflammatory condition was corroborated by a significant decrease in circulating levels of IL-6, a cytokine known to exert anti-inflammatory activities by controlling the level of pro-inflammatory cytokines [78,79].

Intriguingly, the accumulation of AGEs and the imbalance between pro- and anti-inflammatory AGE receptors observed in the liver of KRIT1^+/−^ mice were typical aspects already described in diabetes and obesity, and indicate the possible activation of a low-grade chronic inflammatory response [115,118]. Indeed, it has been widely documented that the AGE/RAGE binding triggers an inflammatory cascade involving TLR4/NFκB and NLRP3 signaling [119,120]. In this regard, it is noteworthy that growing evidence points to a significant impact of loss of KRIT1 function mutations on increased susceptibility to oxy-inflammatory insults, as well as to the substantial contribution of genetic modifiers of antioxidant and anti-inflammatory defenses to the incomplete penetrance and variable expressivity of such mutations [7,8,91].

Remarkably, all the alterations in major regulatory proteins involved in redox/metabolic/AGE pathways detected in the liver of KRIT1^+/−^ mice were recapitulated in KRIT1 knockout cellular models, confirming that they were indeed KRIT1-related events.

## 4. Materials and Methods

### 4.1. Heterozygous KRIT1 Knockout Mouse Model

A heterozygous KRIT1 knockout (KRIT1^+/−^) mouse model on a C57BL/6J background was generated previously by conventional homologous recombination-mediated gene targeting [15]. This animal model mimics the heterozygous state of fCCM patients and has already allowed the identification of systemic effects caused by heterozygous loss-of-function mutations in KRIT1, including enhanced susceptibility to endothelial dysfunction and atherosclerosis [25].

This study was carried out by comparing 26-week-old KRIT1^+/−^ mice (n = 7) and wild-type (WT) littermate controls (n = 7). Mice were housed in a climate-controlled environment at 23 ± 2 °C with artificial day/night cycles (13 h light/11 h dark) and were fed standard rodent chow with free access to water.

Body weight and food/water intake were recorded weekly, whereas fasting plasma levels of glucose were examined monthly. At 26 weeks of age, mice were first subjected to an oral glucose tolerance test (OGTT), and the day after were fasted for 4 h, fully anesthetized with 5% isoflurane via an anesthesia machine (IsoFlo, Abbott Laboratories, Chicago, IL, USA), and then sacrificed by cardiac puncture/exsanguination. Blood samples were collected, and serum was isolated. The liver was rapidly isolated, snap frozen in liquid nitrogen and stored at −80 °C until analysis.

### 4.2. Oral Glucose Tolerance Test

The oral glucose tolerance test (OGTT) was performed after a fasting period of 16 h by administering a glucose solution (2 g/kg) orally via gavage. Once before glucose administration and 15, 30, 60 and 120 min afterwards, blood was collected from the saphenous vein, and glucose concentration was measured with a conventional glucometer (Glucocard G+ meter; Menarini Diagnostics, Florence, Italy), which provides useful approximation of plasma glucose levels.

### 4.3. Plasma Hormones and Cytokines Immunoassay

Plasma levels of insulin, leptin and glucagon hormones, and inflammation-related cytokines, including interleukin-1β (IL-1β), interleukin-6 (IL-6), interferon gamma (IFN-γ) and tumor necrosis factor alpha (TNF-α), were measured using a Bio-Plex Multiplex Immunoassay System (Bio-Rad Laboratories, Hercules, CA, USA), following manufacturer’s instructions.

### 4.4. Glutathione Assay

Reduced and total glutathione were quantified in liver homogenates with the Oxiselect Total Glutathione (GSSG/GSH) Assay Kit (Cell Biolabs Inc, San Diego, CA, USA) following the manufacturer’s instructions.

### 4.5. Quantification of Advanced Glycation End-Products (AGEs) in the Liver

Pentosidine (PEN) and Nε-carboxymethyl-lysine (CML), the most studied markers of AGE accumulation in tissues, were evaluated in total liver extracts after hydrolysis with 0.6 M trichloroacetic acid (C_2_HCl_3_O_2_) and 6 M hydrochloric acid (HCl) for 12 h at 60 °C, using ultraperformance liquid chromatography-tandem mass spectrometry (UPLC-MS), as previously described [121]. Briefly, the chromatographic separation was carried out in an UltiMate™ 3000 HPLC system (Dionex, Milan, Italy) coupled to a high resolution LTQ Orbitrap mass spectrometer (Thermo Scientific, Rodano, Italy) equipped with an atmospheric pressure interface and an electrospray ionization (ESI) source. Samples were analyzed with a Phenomenex Synergi reverse-phase C18 column (dimensions: 150 × 2.1 mm, particle size: 3 μm) at a flow rate of 200 μL/min. A gradient mobile phase composition was adopted: 95/5 to 40/60 in 25 min, 5 mM heptafluorobutanoic acid/acetonitrile. The monitored protonated molecular ions were 205.1188 m/z for CML and 379.2094 m/z for PEN. Quantitative determination of the analytes was done by using PEN and CML calibration data.

### 4.6. In Vitro Model

Mouse embryonic fibroblasts (MEFs) with either KRIT1 homozygous deletion or overexpression in the same genetic background were obtained, as previously described [15,38]. Briefly, KRIT1^−/−^ MEF cells were isolated from KRIT1^−/−^ E8.5 mouse embryos generated by homologous recombination, and a spontaneously immortalized KRIT1^−/−^ MEF cell line (K^−/−^ MEFs) was obtained using the 3T3 protocol. K^−/−^ MEF cells were then infected with a lentiviral construct encoding KRIT1 (pCCLsin.PPT.PGK.KRIT1.Wpre) to restore KRIT1 expression, obtaining a KRIT1-overexpressing MEF cell line (K9/6 MEFs) [15,38]. Cells were cultured at 37 °C and 5% CO_2_ in Dulbecco’s modified Eagle’s medium (DMEM) supplemented with 10% fetal calf serum (FCS), 2 mM glutamine, and 100 U/mL penicillin/streptomycin.

### 4.7. Protein Extraction

Liver proteins were extracted as previously described [122]. Specifically, total proteins were obtained from 10% (*w*/*v*) mice liver homogenates in RIPA buffer (50 mM Tris-HCl, pH 7.5, 150 mM NaCl, 0.5% Nonidet P-40, 0.5% sodium deoxycholate, 0.1% SDS, 10 mM EDTA, and protease inhibitors). After 40 min of incubation in ice, samples were sonicated and centrifuged at 15,000× g at 4 °C for 40 min. Supernatants were collected and stored at −80 °C until use.

Cytosolic and nuclear proteins were extracted from livers homogenized at 10% (*w*/*v*) in a homogenization buffer containing 20 mM HEPES (pH 7.9), 1 mM MgCl_2_, 0.5 mM EDTA, 1% Nonidet P-40, 1 mM EGTA, 1 mM DTT, 0.5 mM PMSF, 5 μg/mL aprotinin, 2.5 μg/mL leupeptin and 2 mM NaVO_3_. Homogenates were centrifuged at 1000× g for 5 min at 4 °C. Supernatants were centrifuged at 10,000× g at 4 °C for 40 min to isolate the cytosolic fraction. The pelleted nuclei were resuspended in extraction buffer containing 20 mM HEPES (pH 7.9), 1.5 mM MgCl_2_, 300 mM NaCl, 0.2 mM EDTA, 20% glycerol, 1 mM EGTA, 1 mM DTT, 0.5 mM PMSF, 5 μg/mL aprotinin, 2.5 μg/mL leupeptin and 2 mM NaVO_3_, and incubated on ice for 30 min for high-salt extraction, followed by centrifugation at 15,000× g for 20 min at 4 °C. The resulting supernatants were collected and stored at −80 °C until use.

Protein extraction from cultured cells was performed as previously described [16,22]. Briefly, cells grown to confluency on plastic petri dishes were washed with ice-cold Tris-buffered saline (TBS) and scraped in ice-cold RIPA lysis buffer containing protease inhibitors. Cell suspensions were then collected in microfuge tubes and incubated at 4 °C for 20 min with constant agitation. Cell lysates were then centrifuged at 15,000× g for 20 min at 4 °C and supernatants were collected in fresh tubes and either analyzed immediately or stored at −80 °C until analysis. Protein content quantification was determined using the Bradford assay (Bio-Rad, Hercules, CA, USA).

### 4.8. Western Blotting Analysis

Equal amounts of proteins were separated by SDS-PAGE and electro-transferred to nitrocellulose or PVDF membranes (GE-Healthcare Europe, Milano, Italy). Membranes were then probed with primary antibodies, listed in Supplementary Table S1, diluted in TBS containing 2% nonfat dry milk and 0.05% Tween, followed by incubation with appropriated horseradish peroxidase (HRP)-conjugated secondary antibodies diluted in TBS containing 2% nonfat dry milk and 0.05% Tween. Protein bands were detected using the Clarity Western ECL substrate (Bio-Rad) and the ChemiDoc™ Imaging System (Bio-Rad, Hercules, CA, USA), and quantified by densitometric image analysis software (Image Lab; Bio-Rad). Results were normalized with respect to the densitometric values of internal loading controls, including β-actin or α-tubulin bands for total and cytosolic extracts, and proliferating cell nuclear antigen (PCNA) bands for nuclear extracts, and then expressed as fold of control mice value.

### 4.9. Glyoxalase-1 (Glo-1) Activity Assay

Glo-1 enzymatic activity in mouse liver total protein extracts was assayed by spectrophotometry, according to the method of McLellan et al. [123]. Briefly, Glo-1 activity was determined by measuring the increased absorbance at 240 nm due to the formation of S-D-lactoylglutathione, using a BioTek Synergy microplate spectrophotometer (BioTek, Winooski, VT, USA). The assay mixture contained 8 mM methylglyoxal (MG) and 2 mM GSH in a sodium phosphate buffer (50 mM, pH 7.4) and a final volume of 1 mL, and was incubated at 37 °C for 10 min before the addition of the protein extract (20–30 μg) to ensure the equilibration of hemithioacetal formation.

### 4.10. Chemical Reagents

Unless otherwise specified, all chemical reagents and media supplements were of analytical grade and purchased from Sigma-Aldrich, and were used as received without any further purification.

### 4.11. Statistical Analysis

The Shapiro-Wilk test was used to assess the normality of the variable distributions. A two-tailed unpaired t-test was adopted for comparison between WT and KRIT1^+/−^ mouse groups. Data were expressed as mean ± SEM (standard error of the mean). Statistical tests were performed with GraphPad Prism 7.0 software package (GraphPad Software, San Diego, CA, USA). The threshold for statistical significance was set to p < 0.05.

## 5. Conclusions

**Figure 8:**
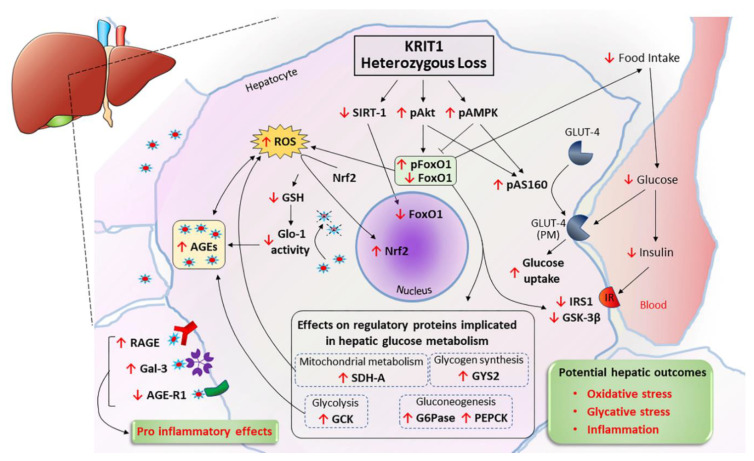
Schematic representation of the main effects of KRIT1 haploinsufficiency detected in the mouse liver. KRIT1 heterozygous loss results in the downregulation of FoxO1 expression/activity, which is likely attributable to substantial changes in three major regulators of FoxO1 nuclear/cytoplasmic shuttling and transcriptional activity, including enhanced activating phosphorylation of Akt and AMPK serine/threonine kinases, and reduced expression of Sirt1 deacetylase. In turn, activated Akt and AMPK promote the activation of AS160, which is required for GLUT4 translocation of the plasma membrane and GLUT4-mediated glucose uptake, whereas FoxO1 downregulation affects both ROS homeostasis and key markers of major hepatic metabolic processes, including gluconeogenesis (G6Pase, PEPCK), glycolysis (GCK), mitochondrial electron transport chain and energy metabolism (SDH-A), and glycogen synthesis (GYS2). As a likely consequence to these effects, the liver of KRIT1 haploinsufficient mice displays also a sustained activation of the master antioxidant transcription factor Nrf2, a reduction of GSH levels and GSH-dependent activity of the AGE-detoxifying enzyme Glo-1, an accumulation of AGEs, and an abnormal expression of AGE receptors, including upregulation of the pro-inflammatory RAGE and Gal-3, and downregulation of the anti-inflammatory AGE-R1. Furthermore, such effects are associated with an impairment of food intake, systemic glucose disposal, and plasma levels of insulin, as well as with the impaired activation of key regulatory proteins involved in insulin signaling, such as IRS1 and GSK3β. Overall, these effects promote oxidative and glycative stress and inflammatory responses.

Overall, our work highlights new, unsuspected roles for the KRIT1 gene in the liver, showing that KRIT1 proper expression and functional interaction with key downstream regulatory proteins, such as FoxO1, is required for the precise and effective regulation of the complex system of interconnected metabolic and redox functions, and antioxidant and antiglycative defenses of the major metabolic organ in the body (Figure 8).

Taken together, our findings have significant implications. Indeed, by expanding the repertoire of KRIT1 functions and not restricting them to the brain vasculature, and providing the first evidence for a major regulatory role of KRIT1 in metabolic and redox functions of the liver, our study opens up new avenues for future research aimed at deep and comprehensive identification and characterization of the underlying molecules and molecular mechanisms. In particular, further studies are needed to unravel the precise mechanisms linking KRIT1 to the expression and activity of the regulatory proteins involved in hepatic glucose metabolism herein analyzed. Indeed, we are aware that our extensive research approach has a few limitations, including the fact that some results are only suggestive of potentially involved molecular pathways, being mainly based on protein expression analysis, and do not provide definitive mechanistic insights. However, our innovative findings pave a new way to expand the mechanistic scenarios underlying the biological functions of KRIT1, and foster deeper investigations to substantiate and extend its novel implications in hepatic glucose metabolism and antioxidant/antiglycative defenses through specifically focused studies. Notably, as KRIT1 expression has been recently reported to be upregulated in liver tumor cells compared to normal primary liver cells [124], our results suggest that KRIT1 might also play a role in hepatic tumor metabolism.

In addition, our findings also raise the intriguing possibility that KRIT1 mutation carriers are predisposed to both CCM disease and metabolic comorbidities, opening the way for future preclinical and clinical studies. In particular, it would be worth testing the possibility that KRIT1 mutation carriers are susceptible to alterations in hepatic and systemic glucose homeostasis, as suggested by our studies in the mouse model. Conversely, it would be interesting to address whether a worsening of hepatic metabolic and redox alterations caused by KRIT1 haploinsufficiency, including an increase in the low-grade hepatic and systemic inflammatory state consequent to the dysregulated hepatic glucose metabolism and inefficient antioxidant/antiglycative defenses, may represent a risk factor for CCM disease pathogenesis.

## Figures and Tables

**Table 1:** **Physiological parameters of KRIT1^+/−^ mice and WT littermate controls.** (1) body weight at 26 weeks of age; (2) average daily food intake during 22 weeks of experimental protocol; (3–6) fasting plasma glucose, leptin, insulin, and glucagon levels at the end of experimental protocol. Data are means ± SEM of 7 mice per group. * p < 0.05; ** p < 0.005; *** p < 0.001 vs. WT.

	Physiological Parameters	WT	KRIT1*^+^*^/*−*^
(1)	Body weight (g)	28.1 ± 1.7	23.8 ± 1.7 *
(2)	Food intake (g/day)	3.25 ± 0.19	2.73 ± 0.17 ***
(3)	Fasting blood glucose (mg/dL)	123.0 ± 5.2	100.7 ± 6.6 *
(4)	Fasting plasma leptin (ng/mL)	4.91 ± 1.02	2.43 ± 0.67
(5)	Fasting plasma insulin (ng/mL)	3.16 ± 0.24	1.69 ± 0.19 **
(6)	Fasting plasma glucagon (ng/mL)	1.35 ± 0.29	1.85 ± 0.22

**Table 2:** **Plasma levels of inflammatory cytokines.** Concentrations of interleuchin-1 β (IL-1β), interleuchin-6 (IL-6), interferon-γ (IFN-γ), and tumor necrosis factor-α (TNF-α) evaluated in plasma of wild-type (WT) and heterozygous KRIT1 knockout (KRIT1^+/−^) mice by Bioplex immunoassay (BioRad). Data are means ± SEM of 7 mice per group. * *p* < 0.05 vs. WT.

	WT	KRIT1 *^+^*^/*−*^
IL-1β (pg/mL)	2.24 ± 0.24	2.69 ± 0.24 *
IL-6 (pg/mL)	7.01 ± 0.95	5.06 ± 0.67 *
IFN-γ (pg/mL)	2.34 ± 0.27	1.98 ± 0.31
TNF-α (pg/mL)	10.70 ± 0.64	14.33 ± 1.33 *

## Data Availability

The data that support the findings of this study are available from the corresponding authors, RM and SFR, upon reasonable request.

## Supplementary Materials

The following supporting information can be downloaded at: https://www.mdpi.com/article/10.3390/ijms231911151/s1.

## Abbreviations

AGE-R1: AGE-receptor-1; AGEs, advanced glycation end-products; Akt, protein kinase B; AMPK, AMP-activated protein kinase; AS160, Akt substrate of 160 kDa; CCM, Cerebral Cavernous Malformation; CML, Nε-carboxymethyl-lysine; FoxO1, forkhead box O1; G6Pase, glucose 6-phosphatase; Gal-3, galectin-3; GCK, glucokinase; GLUT4, glucose transporter 4; Glo-1, glyoxalase-1; GSH, glutathione; GSK3β, glycogen synthase kinase 3β; GYS2, glycogen synthase-2; IFN-γ, interferon gamma; IL-1β, interleukin-1β; IL-6, interleukin-6; IR, insulin receptor; IRS1, insulin receptor substrate 1; KRIT1, Krev interaction trapped 1; MEFs, mouse embryonic fibroblasts; NFκB, nuclear factor-kappa B; NLRP3, NLR family pyrin domain containing 3; Nrf2, nuclear factor erythroid 2-related factor 2; PCNA, proliferating cell nuclear antigen; PEN, pentosidine; PEPCK, phosphoenolpyruvate carboxykinase; PKC, protein kinase C; RAGE, receptor for AGE; ROS, reactive oxygen species; SDH-A, succinate dehydrogenase A; Sirt1, Sirtuin 1; SOD2, superoxide dismutase 2; TLR4, Toll-like receptor 4; TNF-α, tumor necrosis factor alpha; WT, wild-type.
