# Supplementary material for: Heterozygous Loss of KRIT1 in Mice Affects Metabolic Functions of the Liver, Promoting Hepatic Oxidative and Glycative Stress
Source: Int J Mol Sci. 2022 Sep 22;23(19):11151. doi: 10.3390/ijms231911151 (PMC9570113; doi:10.3390/ijms231911151)
Supplement: Supplementary file 1 [file ijms-23-11151-s001.zip › ijms-1887588-supplementary.pdf]

Supplementary Materials

Supplementary Table S1. Antibodies used for Western blot analysis.

**Target protein**	**Host species**	**Antibody supplier**	**Catalog Number**	**Dilution**
AGE-R1	Rabbit	Gene Tex	GTX102211	1:500
Akt	Rabbit	Cell Signaling Technology	#9272	1:1000
AMPKα	Rabbit	Cell Signaling Technology	#2532	1:1000
AS160	Rabbit	Cell Signaling Technology	#2447	1:1000
FoxO1	Rabbit	Cell Signaling Technology	#2880	1:1000
G6Pase	Mouse	Abcam	ab243319	1:500
Galectin-3	Mouse	Affinity BioReagents	MA1-940	1:1000
GCK	Mouse	Santa Cruz Biotechnology	sc-17819	1:500
Glo-1	Rabbit	Gene Tex	GTX105792	1:500
Glycogen synthase-2	Mouse	Santa Cruz Biotechnology	sc-390391	1:500
GSK3β	Rabbit	Santa Cruz Biotechnology	sc-9166	1:500
KRIT1	Rabbit	Abcam	ab196025	1:1000
IRS	Mouse	Cell Signaling Technology,	#3194	1:1000
Nrf2	Rabbit	Thermo Scientific	PA5-27882	1:500
pAkt-Ser473	Rabbit	Cell Signaling Technology,	#9271	1:1000
pAMPKα-Thr172	Rabbit	Cell Signaling Technology,	#2531	1:1000
pAS160-Ser588	Rabbit	Cell Signaling Technology,	#8730	1:1000
PCNA	Mouse	Santa Cruz Biotechnology	sc-56	1:1000
PEPCK	Mouse	Santa Cruz Biotechnology	sc-271029	1:500
pFoxO1-Ser256	Rabbit	Cell Signaling	#9461	1:1000
pGSK3β-Ser9	Goat	Santa Cruz Biotechnology	sc-11757	1:500
pIRS-Ser307	Rabbit	Cell Signaling Technology,	#2381	1:1000
RAGE	Mouse	Santa Cruz Biotechnology	sc-365154	1:500
SDH-A	Mouse	Santa Cruz Biotechnology	sc-377302	1:500
SIRT1	Rabbit	Santa Cruz Biotechnology	sc-15404	1:500
α-tubulin	Mouse	Abcam	ab7291	1:2000
β-actin	Mouse	Santa Cruz Biotechnology	sc-47778	1:1000
